# Fast Food, Health Impacts, and Policy Responses in China and India

**DOI:** 10.5334/aogh.5264

**Published:** 2026-07-22

**Authors:** Andrew Williams

**Affiliations:** 1 Undergraduate Student at Boston College, Chestnut Hill, MA, USA

**Keywords:** China, India, fast food, non-communicable diseases, health impacts, policy, obesity, diabetes, hypertension, regulations

## Abstract

*Background:* In China and India, the fast-food industry has rapidly expanded in the past three decades, alongside rising rates of overweight, obesity, and diet-related non-communicable diseases (NCDs). Both governments have implemented policy responses addressing fast-food-related health risks.

*Objectives:* (1) Synthesize research on fast food’s health impacts in China and India to assess the existing evidence base and compare reported health outcomes. (2) Compare the structure, scope, and effectiveness of policy responses in the two countries.

*Methods:* A narrative review of peer-reviewed studies since 2010 was conducted. Studies were selected based on relevance to fast-food-related health outcomes and policy responses in China and India. In total, 52 peer-reviewed studies were selected. Grey literature was also used to document recent policy developments.

*Results:* Health outcomes: China’s evidence base includes longitudinal and quasi-causal studies, whereas India relies largely on large population analyses and school-based studies. In China, studies link fast-food exposure to childhood and adult obesity, cardiometabolic risks, and acute health outcomes. In India, studies report similar associations, including hypertension and diabetes. Marginalized groups, including migrant workers and pregnant women, are particularly vulnerable, reinforcing health inequities.

Policy effectiveness: National policies in both countries prioritize education over binding regulation. Their effectiveness against fast-food consumption and diet-related NCDs is largely limited and, especially in India, under-researched. Regulations on fast-food sales, advertising, and mandatory front-of-package nutrition labels are either absent or face significant implementation challenges. China’s centralized framework, Healthy China 2030, contrasts with India’s more fragmented approach, which features multiple frameworks and some regulatory measures beyond education. Binding subnational interventions, including zoning laws and health taxes, have demonstrated effectiveness in reducing fast-food consumption.

*Conclusions:* Educational policies alone are insufficient to curb the growing health impacts of fast-food consumption. Stronger, enforceable national regulations are needed to reshape food environments and reduce the growing burden of fast-food-related NCDs.

## Introduction

China and India, the world’s two most populous countries, have experienced rapid expansion of fast food consumption in the past three decades [1, 2]. Once marginal in both settings, fast food has become deeply embedded in urban food environments and dietary patterns. Fast food’s spread has coincided with sharp increases in diet-related non-communicable diseases (NCDs), exacerbating the double burden of malnutrition: the existence of undernutrition alongside overweight, obesity, and metabolic disease [3, 4].

In China, the first major Western fast-food chain to open was KFC in 1987, and fast-food growth then peaked from the mid-1990s through the 2000s [5]. From 1997 to 2006, the number of annual transactions at fast-food restaurants increased fivefold, and the total market value of fast food increased ninefold [6]. From 1999 to 2013, fast-food revenue maintained a very high average annual growth rate of 18%; the current rate remains high at 6%–7% [7]. In India, growth occurred later. In 1996, McDonald’s, KFC, and Pizza Hut all opened their first Indian locations, and rapid industry growth followed from the mid-2000s into the 2020s. In 2025, the industry’s annual growth rate was estimated at 30–40% [8].

In both countries, studies have linked fast-food consumption to overweight and obesity among children and adults [1, 9–13], as well as to hypertension and type-2 diabetes [14–17]. In China, several studies have employed quasi-experimental methods to strengthen causal inference [1, 10]. Still, this evidence remains fragmented, context-specific, and rarely synthesized in a comparative framework.

China and India together account for a substantial share of the global NCD burden: China has the highest absolute numbers of overweight, obesity (defined per WHO criteria as body mass index (BMI) ≥25 and ≥30, respectively), and type-2 diabetes cases globally, followed by India [18, 19]. China’s combined overweight and obesity rate among adults is 57%, which is 14% higher than the global average of 43% [20, 21]. In India, the combined rate increased by more than twofold from 1990 to 2022, rising from 7% to 16%, whereas the global average increased by less than twofold over the same period, from 25% to 43% [22].

China and India’s governments have implemented a range of policy responses to address fast-food-related health risks, including education campaigns, regulations on sales and advertising, and clinical screening and management programs. China’s policies are streamlined into a single framework and are more focused on broad educational campaigns, while India’s policies are roughly divided into two national frameworks and include more direct, on-the-ground interventions such as population-level screenings, NCD clinics, and mandated advertisements against junk food. Both countries have enacted fast-food advertising restrictions, though these vary in enforceability and specificity. At the subnational level, China’s province-level zoning laws and Kerala’s junk food tax have shown promise, and each country is moving toward mandatory unhealthy-food labeling on processed foods. While studies in both countries have assessed the enforceability and effectiveness of specific policies [23–26], this review offers a holistic comparison of national and subnational responses to identify which approaches have been most and least effective in curbing fast-food-related NCDs.

By comparing health outcomes and state action in China and India, this review identifies key similarities, differences, evidence gaps, and policy outcomes, thereby providing insight into how fast-food-related health risks are manifesting and being addressed in two of the world’s most influential emerging economies.

## Methods

This study employed a narrative literature review design, appropriate given the heterogeneity of study designs and policy contexts across China and India. An extensive search was conducted in PubMed, Google Scholar, ProQuest, ScienceDirect, Scopus, and JSTOR. Keywords frequently used were “China,” “India,” “fast food,” “ultra-processed food,” “health,” “obesity,” “diabetes,” “non-communicable diseases,” “government,” and “policy.” Peer-reviewed articles published since 2010 were selected based on relevance to the review objectives and methodological transparency. In total, 52 peer-reviewed studies were included.

Following established narrative review methodology, studies were organized thematically by subtopic and chronologically within each theme. Studies were interpreted to draw conclusions about individual findings and to identify patterns, similarities, and differences across the literature. Reference lists of included articles were also reviewed to identify additional relevant sources [27].

The available literature specifically examining the health impacts of fast food was limited. Much of the existing evidence on diet-related health outcomes focuses on ultra-processed food exposure more broadly, with fast food addressed as a subcategory of unhealthy food consumption rather than as a discrete exposure. A similar pattern was observed in the policy literature: neither China nor India has enacted national policies targeting fast food exclusively, and broader regulatory and educational frameworks addressing unhealthy foods represent de facto governmental responses to fast-food-related health impacts. Accordingly, studies on ultra-processed food and unhealthy food consumption were included if they were methodologically comparable to fast-food-specific studies: that is, they employed similar sample sizes, measured similar health outcomes, and used comparable analytical approaches such as correlation-based or longitudinal designs. Of the studies examining health impacts, 5 did not research fast food specifically. Of the studies examining policy, 16 did not research fast food specifically, although 4 of these examined foods high in fat, sugar, and salt (HFSS) sold by corporate vendors, which are functionally equivalent to fast food.

Peer-reviewed literature on national and subnational food-environment policies introduced within the past 2 years was limited. To capture recent governmental actions, grey literature was reviewed, including official government announcements and reporting by established news outlets. News sources were used solely to document factual policy developments (e.g., enactment dates and scope of regulations) and not to infer policy effectiveness.

Definitions of overweight and obesity varied across studies. Although lower BMI thresholds are sometimes applied in Asian populations, most studies included here defined overweight as BMI ≥ 25 kg/m² and obesity as BMI ≥ 30 kg/m²; these definitions are used throughout unless otherwise specified.

## Results

### Fast-food impacts on Chinese health

#### Obesity and excessive weight gain in children and students

In China, five studies found links between fast-food consumption and obesity in children and students. A landmark 2018 study provided quasi-causal evidence that fast-food consumption causes pediatric obesity [1]. Using 7 years of nationally representative health survey data combined with restaurant density data from major fast-food chains, the authors estimated that increases in fast-food restaurants accounted for approximately 15% of the rise in childhood overweight and obesity. Specifically, the presence of a nearby fast-food restaurant was associated with a 6-percentage-point higher likelihood of overweight or obesity. Additionally, two studies with sample sizes of tens of thousands of children found significant associations between fast-food consumption and obesity (Shan’s study classifies overweight as BMI ≥ 24 kg/m² and obesity as BMI ≥ 28 kg/m²) [9, 28]. Finally, two studies published in 2025 highlighted the role of broader environmental influences on obesity: One reported that parental fast-food consumption was associated with obesity in their children [29], while the other found that increased internet use was linked to higher obesity risk, with fast-food preferences identified as a significant mediating factor (overweight being BMI ≥ 24 kg/m² and obesity being BMI ≥ 28 kg/m²) [30].

#### Obesity and excessive weight gain in adults

Three studies link fast-food consumption with excessive body mass in Chinese adults [10, 11, 31]. A 2021 study found that among female Filipina migrant workers in Macao, “Fast food restaurant density within a 0.5-mile buffer zone around one’s home was significantly associated with a 7% increase” in overweight or obesity [10]. This quasi-causal study, comparable to the 2018 pediatric obesity study discussed above, provides additional evidence that fast-food restaurant proximity is associated with obesity. Furthermore, two studies with sample sizes of ≥4,000 adults reported similar associations: one linked fast-food restaurant presence to increased waist-to-height ratio and waist-to-hip ratio, while the other associated eating out to overweight [11, 31]. Notably, both studies predate 2014, highlighting a gap in up-to-date large-sample evidence. These adult studies, along with the pediatric studies above, provide evidence that fast food has caused excessive weight gain in many Chinese demographics and locations.

#### Adverse metabolic outcomes

Two 2025 studies linked fast-food consumption to adverse metabolic outcomes in Chinese participants [14, 15]. One evaluated body composition of 1,375 obese children and found that fast-food consumption was a significant determinant for metabolically unhealthy obesity (MUO) caused by either hypertension, hyperglycemia, or dyslipidemia (overweight being BMI ≥ 24 kg/m² and obesity being BMI ≥ 28 kg/m²) [14]. The other study found that among 306 pregnant women, fast-food consumption was a risk factor for excessive gestational weight gain (GWG). Excessive GWG poses cardiometabolic risk, as mothers may develop gestational diabetes, hypertension, and preeclampsia, and offspring may experience hyperglycemia [15].

#### Acute health outcomes

One 2023 study links fast-food consumption to acute health outcomes [32]. Conducted among 4,058 participants in Northeast China, it found a correlation between fast-food consumption and upper respiratory tract infections. This, along with the studies of cardiometabolic risks, provides evidence that fast food jeopardizes Chinese health in ways that extend beyond weight gain.

### Fast-food impacts on Indian health

#### Obesity and excessive weight gain in children and students

In India, five school-based studies have found associations between fast-food consumption and excessive weight gain in children [12, 33–36]. Three recent 2025 studies, each analyzing hundreds of students across one or more schools, reported direct links between fast-food consumption and markers of overweight, including increased central adiposity, BMI, and obesity [33–35]. Earlier studies from 2015 similarly reported associations between fast-food consumption and obesity in school populations (Nitin’s study defines obesity as BMI ≥ 28 kg/m²) [12, 36].

#### Obesity and other forms of excessive weight gain in adults

A nationally representative 2023 study including 250,000 Indian adults across tribal, rural, and urban sites found that unhealthy diets and ultra-processed-food consumption, a category that includes fast food as well as packaged snacks and meals, were the predominant contributors to obesity (BMI ≥ 25 kg/m²) [13].

#### Adverse metabolic outcomes

Studies have proven associations between fast food and ultra-processed food (UPF) consumption in India and adverse metabolic outcomes, including hypertension, diabetes, visceral fat accumulation, and micronutrient deficiencies [16, 17, 37]. A 2025 study of 1,000 working adults found that those who consumed more food prepared away from home—typically high in calories, fat, and sodium—were more likely to have hypertension, diabetes, and chronic respiratory disease, after adjusting for other risk factors [16]. Additionally, a 2025 study found that among 110 university students, higher UPF consumption “was significantly associated with greater visceral fat accumulation” [37]. Regarding nutritional outcomes, a 2023 study of 589 adults found that ultra-processed food consumption was associated with niacin and folate deficiencies [38]. According to a 2025 study published in the *Journal of Economics, Finance and Accounting Studies*, two other studies found relationships between fast food and cardiometabolic risks [17]. One study found that participants “who consumed fast food more than four times a week had a 28% higher prevalence of hypertension and a 27% higher rate of type-2 diabetes.” The other found that among young adults in two states, “the entry of fast-food chains coincided with a marked increase in obesity and prediabetes.” Notably, these two studies could not be independently located.

#### Acute health outcomes

Studies found that fast-food consumption in India is associated with acute gastrointestinal illness [38–40]. A 2025 study found that among 430 1–5 year olds, fast-food consumption was associated with acute diarrheal disease [39]. In addition, a 2025 study found that among 303 medical students, 28.7% reported gastrointestinal discomfort after online meals [40]. Like China, a breadth of studies in India finds relationships between fast-food consumption and multiple acute and chronic, metabolic conditions.

### Comparing governmental responses to fast-food-related health impacts

#### Few national, binding policies in either country

Both China and India have very few national, binding policies that directly limit fast-food consumption. In China, no national, binding policies specifically limiting fast-food sales or marketing have been identified in the literature, government regulations, or public health reports [41, 42]. India has passed several laws banning the sale and advertising of unhealthy foods, particularly in and around schools, but these laws are frequently ignored or weakly enforced [24, 26, 43, 44]. As a result, in both countries, national responses rely primarily on education-based frameworks rather than enforceable regulation.

#### Comparison of national frameworks

China and India seek to curb fast-food-related NCDs through national, multiyear policy frameworks that prioritize education and behavior change over binding restrictions. In both countries, public education forms the backbone of national strategies. This includes dietary guidelines, health literacy campaigns, and physical activity promotion. Notably, the countries’ policy structures differ substantially. China relies on a centralized, unified national framework, while India’s response is more fragmented, consisting of multiple programmatic initiatives embedded within existing institutions. Although overarching goals are similar, the degree of coordination and policy coherence is greater in China [45, 46, 47].

#### Healthy China 2030

China’s response to rising fast-food consumption and NCDs is highly centralized within Healthy China 2030; few major policies exist outside this framework. Approved in 2016, Healthy China 2030 positioned health as a national development priority and articulated long-term targets, including reducing childhood overweight and obesity growth rates [45].

Following its approval, Healthy China 2030 expanded population-wide health education through aligned initiatives. In 2017, the “Three Reductions and Three Health Benefits” campaign promoted reduced intake of salt, oil, and sugar, while the Health Action Plan for All (2017–2025) emphasized disease prevention, health literacy, and equity, reinforcing HC2030’s emphasis on individual behavior change [48, 49].

From 2019 onward, China focused on strengthening administrative coordination and encouraging private sector cooperation rather than introducing regulatory tools [50]. In 2019, China adopted its first comprehensive public health law, which codified the government’s responsibility to protect public health [51]. Also, the 14th Five-Year Plan (2021–2025) aimed to increase health insurance coverage, healthcare access, and public health research [52]. Finally, annual implementation reports outlined Healthy China 2030 priorities.

In 2024, China adopted a more targeted, visible approach through a 3-year National Obesity Campaign, which introduced weight management clinics, obesity-focused educational materials, and the installation of scales in many hotels. Although still largely educational, this campaign marked a shift toward action-oriented implementation [21].

#### India’s national frameworks

Unlike China’s centralized approach, India has developed two distinct national frameworks to address fast-food-related NCDs, reflecting its decentralized governance structure [46, 53]. Both frameworks were created after Healthy China 2030, which is consistent India’s later rise of fast-food consumption and NCD prevalence. While education remains central, India’s frameworks incorporate more direct, on-the-ground interventions than China’s national strategy.

One of India’s two frameworks, the National Programme for Non-Communicable Diseases, launched in 2023. Like Healthy China 2030, the National Programme for Non-Communicable Diseases emphasizes health education and surveillance, but also includes population-level screening of adults aged 30 years and older and NCD clinics in hospitals [46].

India’s second major framework, Eat Right India, was introduced in 2018 by the Food Safety and Standards Authority of India (FSSAI). Eat Right India prioritizes nutrition education through measures such as mandatory “Oil and Sugar Boards” in public spaces [47] and has taken actions beyond education, including a nationwide trans-fat limit of <2% [54], voluntary industry commitments to limit advertising to children, and a certification program covering over 4,000 healthy food vendors [53].

Both countries’ national frameworks rely on education and voluntary commitments rather than regulations. Notably, India’s frameworks incorporate more action-based strategies alongside education, although China’s recent National Obesity Campaign signals a shift toward more direct interventions against diet-related NCDs.

#### Comparing effectiveness of national frameworks

Evidence indicates that Healthy China 2030 has not effectively curbed fast-food-related NCDs [23]. Shortly after its introduction in 2016, modeling analyses predicted that China would face “daunting challenges” in meeting Healthy China 2030 overweight and NCD targets [3, 55]. More recent scholarship supports that China’s response to the obesity crisis has been inadequate: Although one study reported improvements in general population health under Healthy China 2030 [56], another specifically examining obesity and diet-related NCDs found that China’s national response has been “inadequate” [23].

In India, no studies directly evaluate or predict the effectiveness of the National Programme for Non-Communicable Diseases or Eat Right India, representing a significant literature gap. It is worth noting that the National Programme for Non-Communicable Diseases only began in 2023, so policy analysis may soon evaluate its effectiveness. However, Eat Right India began in 2018, and there is no literature evaluating its efficacy. Obesity and diet-related NCDs continue to rise, suggesting limited effectiveness, similar to China’s experience [22, 57, 58].

#### Indian bans on fast food near schools: increasingly common but unenforced

In contrast to China, India has introduced multiple binding regulations banning the sale and advertising of unhealthy foods in specific contexts, particularly around schools. However, these policies are widely unenforced [24, 26]. Since 2015, agencies including the FSSAI have passed six national policies restricting HFSS foods near schools. Evidence shows poor compliance: vendors continue selling HFSS foods within school premises, and students are frequently exposed to unhealthy food advertising [43].

In 2020, the FSSAI enacted a landmark ban on the sale and advertising of HFSS foods within 50 meters of schools [44]. Nonetheless, enforcement remained weak: one study found that many vendors still sold HFSS within restricted zones. Many vendors and school staff were unaware of the ban [24].

Although several Indian regulations are intended to restrict unhealthy food advertising, enforcement gaps persist, due to limited awareness, the absence of a uniform definition of HFSS foods, and loopholes allowing advertisements not explicitly “directed” at children [26].

#### Fast-food advertising regulations

Effective regulation of fast-food advertising is limited in both countries. China has national child marketing restrictions that apply to unhealthy food, but these policies do not explicitly or comprehensively restrict fast-food or ultra-processed-food marketing across multiple platforms, resulting in low overall implementation [41]. In India, as discussed, multiple regulations are meant to restrict unhealthy food advertising, but weak enforcement and definitional gaps limit their impact [26].

#### Front-of-package labels

Front-of-package labels (FOPLs) are widely used globally to discourage unhealthy food consumption [59], yet neither China nor India has mandated front-of-package labels. In both countries, experimental evidence indicates that front-of-package labels improve consumers’ ability to identify unhealthy foods, with generally positive effects on purchasing decisions.

China released a draft voluntary national front-of-package labels standard in 2024, scheduled for implementation in 2027. Also, the Chinese food industry has begun adopting front-of-package labels to align with global practices [60]. A large-sample 2025 study found front-of-package labels encouraged healthier purchasing behavior [61].

India is closer to mandating front-of-package labels nationally, following prolonged debate and industry opposition since 2019 [62]. As of 2025, the FSSAI is nearing a mandate [63]. One study found that front-of-package labels improved Indian consumers’ identification of unhealthy foods but did not reduce intentions to purchase such foods [64]. Nonetheless, evidence from China suggests a mandatory front-of-package labels system in India could meaningfully reduce diet-related NCDs.

#### Effectiveness of subnational binding measures

Despite limited national regulation, binding subnational policies have demonstrated effectiveness in both countries [25, 42, 65]. In China, several provinces have implemented zoning restrictions on fast-food outlets, which a multidisciplinary evaluation found to be enforceable and effective [42]. Also, school-level bans on nearby unhealthy food stalls in multiple cities were associated with lower BMI among children [65]. In India, Kerala’s short-lived 14.5% junk food tax in 2016 was associated with a 3.9% reduction in fast-food purchases [25, 66]. As of 2025, India’s Finance Ministry has proposed a broader health tax on ultra-processed foods [67]. These cases highlight the effectiveness of binding subnational measures even when national frameworks rely primarily on education.

## Discussion

This review highlights the substantial health impacts of fast-food consumption in China and India, as well as the persistent shortcomings of national policy. Both countries face rising levels of overweight, obesity, and cardiometabolic risk across children and adults. Evidence from quasi-causal studies in China and large-scale population analyses in India establishes that fast-food consumption contributes directly to these outcomes [1, 10, 13]. Marginalized populations—including female migrant workers and pregnant women who face barriers to adequate healthcare—are disproportionately affected, reinforcing health inequities [10, 15]. While China benefits from a more developed body of longitudinal research, India’s evidence base is rapidly expanding but often relies on older survey data, leaving a gap in understanding more recent trends during the fast-food industry’s rapid growth.

Despite mounting evidence of harm, national responses in both countries remain predominantly educational and nonbinding. Neither country has enforceable regulations on fast-food sales or advertising, nor have they adopted mandatory front-of-package labels, a strategy used in several countries to improve diets, and which has been shown to be effective in experimental studies in China.

China’s centralized Healthy China 2030 framework prioritizes health education and individual behavior change; however, evidence indicates that Healthy China 2030 has thus far been insufficient in reversing national obesity trends [23]. India’s more fragmented, two-framework approach combines education with voluntary industry commitments and targeted interventions. Nonetheless, no policy analysis has yet assessed the effectiveness of either framework, representing a significant research gap. The persistence, and in some cases acceleration, of obesity and diet-related NCD trends suggests that India’s mostly educational strategies have been insufficient to counteract fast-food consumption [57].

In contrast, subnational binding measures, such as zoning restrictions in China and state-level taxes in India, have demonstrated strong enforceability and sizable impacts on consumer behavior [25, 42]. More broadly, dozens of countries have enacted binding laws targeting unhealthy foods and sugar-sweetened beverages, including taxes and advertising restrictions; multinational evidence indicates that these taxes substantially reduce purchases and consumption [68, 69].

In conclusion, the comparative experience of China and India demonstrates that education, while necessary, is insufficient as a standalone strategy to address fast-food-related health risks. Sustained reductions in obesity and diet-related NCDs will require stronger, enforceable national regulations that reshape food environments. These regulations could be effectively complemented by targeted subnational policies along with public education. Without regulatory foundations, efforts to improve population health and reduce health inequities are unlikely to achieve a durable, population-level impact.

## Data Availability

The author had full access to all data and is solely responsible for the content of the manuscript.
